# Medical pre-hospital management reduces mortality in severe blunt trauma: a prospective epidemiological study

**DOI:** 10.1186/cc9982

**Published:** 2011-01-20

**Authors:** Jean-Michel Yeguiayan, Delphine Garrigue, Christine Binquet, Claude Jacquot, Jacques Duranteau, Claude Martin, Fatima Rayeh, Bruno Riou, Claire Bonithon-Kopp, Marc Freysz

**Affiliations:** 1 Université de Bourgogne, Service d'Anesthésie et Réanimation - SAMU 21, Hôpital Général, 3 Rue Faubourg Raines, Centre Hospitalier Universitaire de Dijon, Faculté de médecine, 21033 Dijon Cedex, France; 2 Fédération des Urgences - SAMU59, Centre Hospitalier Régional Universitaire de Lille, Avenue Oscar Lambert, 59037 Lille Cedex, France; 3 INSERM CIE 01, Centre d'Investigation clinique - Epidémiologique clinique du CHU de Dijon, 21033 Dijon Cedex, France; 4 Pôle Anesthésie Réanimation, CHU de Grenoble, 38043 La Tronche Cedex, France; 5 Université Paris Sud-Paris XI, Hôpital Bicêtre, Département d'Anesthésie-Réanimation, Assistance Publique-Hôpitaux de Paris, 94275 Le Kremlin-Bicêtre, France; 6 Université de la Méditerranée, Centre de traumatologie et Département d'Anesthésie Réanimation, Centre Hospitalier Universitaire Nord, Boulevard Pierre Dramard, 13015 Marseille, France; 7 Département d'Anesthésie Réanimation Chirurgicale, Centre Hospitalier Universitaire La Milétrie, rue de la Milétrie, 86000 Poitiers, France; 8 Université Pierre et Marie Curie-Paris 6, Service d'Accueil des Urgences, GH Pitié-Salpêtrière, Assistance Publique-Hôpitaux de Paris, 75013 Paris, France

## Abstract

**Introduction:**

Severe blunt trauma is a leading cause of premature death and handicap. However, the benefit for the patient of pre-hospital management by emergency physicians remains controversial because it may delay admission to hospital. This study aimed to compare the impact of medical pre-hospital management performed by SMUR (Service Mobile d'Urgences et de Réanimation) with non-medical pre-hospital management provided by fire brigades (non-SMUR) on 30-day mortality.

**Methods:**

The FIRST (French Intensive care Recorded in Severe Trauma) study is a multicenter cohort study on consecutive patients with severe blunt trauma requiring admission to university hospital intensive care units within the first 72 hours. Initial clinical status, pre-hospital life-sustaining treatments and Injury Severity Scores (ISS) were recorded. The main endpoint was 30-day mortality.

**Results:**

Among 2,703 patients, 2,513 received medical pre-hospital management from SMUR, and 190 received basic pre-hospital management provided by fire brigades. SMUR patients presented a poorer initial clinical status and higher ISS and were admitted to hospital after a longer delay than non-SMUR patients. The crude 30-day mortality rate was comparable for SMUR and non-SMUR patients (17% and 15% respectively; *P *= 0.61). After adjustment for initial clinical status and ISS, SMUR care significantly reduced the risk of 30-day mortality (odds ratio (OR): 0.55, 95% CI: 0.32 to 0.94, *P *= 0.03). Further adjustments for the delay to hospital admission only marginally affected these results.

**Conclusions:**

This study suggests that SMUR management is associated with a significant reduction in 30-day mortality. The role of careful medical assessment and intensive pre-hospital life-sustaining treatments needs to be assessed in further studies.

## Introduction

According to the World Health Organization, injuries are the leading cause of death for people under the age of 45. In Europe, nearly 800,000 people die from injuries every year [1]. The prevention and management of severe trauma are thus major public health issues in most countries.

Blunt trauma and penetrating trauma present major differences in clinical presentation, management and outcomes [2,3]. In contrast to trauma epidemiology in the United States, blunt trauma is more frequent than penetrating trauma in most European countries, and the leading cause of severe trauma. Medical pre-hospital management (SMUR: Service Mobile d'Urgences et de Réanimation), generally performed by an emergency physician (EP), may take longer than care provided by fire brigades. The benefit for blunt trauma victims of SMUR management on the scene of the accident is controversial [4].

To our knowledge, no previous studies have investigated the benefit of SMUR *versus *non-SMUR management on the prognosis of patients with blunt trauma. The FIRST (French Intensive care Recorded Severe Trauma) observational study was initiated to describe the management of patients with severe blunt trauma. The main aim of the present analysis was to examine whether SMUR care reduced 30-day mortality, in comparison with non-SMUR care provided by fire brigades. A secondary aim was to assess the impact of SMUR care on 72-hour mortality.

## Materials and methods

### Patients

In France, two rescue systems are involved in the pre-hospital management of trauma patients. In the event of an accident, both the fire brigade and pre-hospital medical emergency dispatching centre (SAMU: Service d'Aide Médicale Urgente) can be alerted. A fire brigade is systematically dispatched to the scene. The SAMU may decide to send a pre-hospital medical emergency team (SMUR), either directly, as a result of the phone evaluation of the accident, or later, as a result of the on-scene evaluation by the fire brigade. If there is no request for SMUR support, the fire brigade will provide only basic life support and take the patient to the closest hospital. However, according to French regulations, when the vital signs of a patient transported by the fire brigade cease, the fire brigade vehicle has to stop; the staff has to call the SMUR and begin cardio-pulmonary resuscitation with chest compression and ventilation while waiting for the arrival of the SMUR. The French SAMU/SMUR system is well described in the literature [2]. Briefly, when the SMUR is present on the scene, the EP may decide to refer the patient either to the closest hospital or to a university hospital trauma centre if a major trauma is suspected [5]. Each SMUR unit is staffed by an EP, a nurse and a specially trained ambulance driver. Depending on the clinical assessment of the patient, the emergency physician may initiate early life-sustaining treatment. According to French guidelines, tracheal intubation is systematically performed for patients with severe brain trauma (GCS: Glasgow Coma Scale <8), with respiratory distress syndrome and/or with haemorrhagic shock. Rapid sequence intubation is systematically used for tracheal intubation and sedative infusion is started early to prepare the patient for mechanical ventilation. Fluid infusion is performed on the basis of clinical assessment and mean arterial pressure (MAP). The haemodynamic target depends on whether patients have neurological injury (cerebral and/or medullar) (MAP: 85 to 90 mmHg) or not (MAP: 65 mmHg). In order to achieve the MAP target, the EP uses careful crystalloid infusion. If the initial MAP is very low (mean arterial pressure <60 mmHg), patients receive a bolus infusion of colloid and/or hypersaline solution. Continuous norepinephrine infusion can start quickly for patients with initial collapse, or in order to limit an excess of fluid loading. According to French guidelines, it is necessary to begin continuous norepinephrine infusion when fluid loading exceeds 2,000 ml and/or after beginning sedative infusion. Mannitol may be used if clinical intracranial hypertension exists or appears. Medical monitoring includes invasive arterial blood pressure, the use of continuous capnometry and on-board arterial blood gas measurement [6]. The patient's response to treatment is used by the dispatching physician to determine the most appropriate facility for the patient [7].

The FIRST prospective study involved intensive care units (ICU) and emergency departments from 14 university hospitals located throughout France. University hospitals in France correspond to Level 1 trauma centres in the United States. Between December 2004 and March 2007, study centres were asked to record data regarding consecutive patients with severe blunt trauma in a computerized and anonymous database. Inclusion criteria were age (18 years or over) and severe blunt trauma, defined as trauma requiring admission into a university hospital ICU within 72 hours after injury or, in the case of early death before ICU admission, trauma managed by SMUR units from university hospitals. Exclusion criteria were penetrating traumas, and deaths occurring before the implementation of any advanced life-sustaining treatment. The latter condition means that trauma patients managed by fire brigades who died before admission to an ICU were not included in the study. A total of 3,205 patients were eligible for inclusion in the FIRST study.

After checking for data quality, patients with either incomplete (*n *= 97) or poor quality data (aberrant or illogical data, *n *= 281) regarding hospital of first admission, injury severity score (ISS) and vital status were subsequently excluded. Patients with unknown information about SMUR/non-SMUR management (*n *= 124) were also excluded. Thus, the present analysis was restricted to 2,703 patients suffering from severe trauma, alive upon arrival at the hospital, for whom complete and high quality data on the major variables of interest were available. According to French law (law 88-1138 relative to Biomedical Research of 20 December 1988 modified on 9 August 2004), this non-interventional study did not require approval by an Ethics Committee nor informed signed consent from patients. The study was declared to, and approved by, the National Commission for Data Processing and Civil Liberties (authorization n° 05-1059 obtained on 24 February 2005). However, according to French law, all patients or their families were informed about the study by the ICU physician[8].

### Data collection

ICU physicians collected data from the medical records of SMUR units, emergency divisions and ICUs, regardless of the hospital of first admission. In each centre, ICU physicians entered data into the FIRST database with the help of local research assistants. The eligibility criteria were checked online by the research assistants of the Coordination Centre in Dijon. Every month, the Coordination Centre extracted data for quality control. In cases of missing, aberrant or illogical mandatory data, queries were sent to local research assistants. At the end of the inclusion period, data monitoring was performed by the Coordination Centre in order to validate data quality on a random sample of 7% of patients. Unreliable variables were discarded from the analysis.

ICU physicians collected the following data: 1 - patient characteristics; 2 - data about accident circumstances, condition of victims in traffic-related accidents, and rescue services mobilized for patient transport (SMUR units or fire brigade units); 3 - hospital units involved in early care of the patient before admission to the ICU; 4 - clinical and biological data on the pre-hospital phase, if available, at first hospital admission and at 24 h and 72 h after trauma; 5 - a summary of clinical variables at patient discharge or death.

During the pre-hospital phase, the following data were recorded for SMUR patients: initial physiological variables (arterial pressure, respiratory rate, SpO_2_), pupil status, GCS and life-sustaining treatments (venous line, fluid loading and catecholamine administration, tracheal intubation, ventilation, blood products, chest tube).

For all patients, information on physiological variables and life sustaining treatments was also collected upon arrival at the first hospital, and 24 h and 72 h after the injury. The first available measurement, either at the pre-hospital phase or upon hospital admission, was used to describe the initial physiological status of the patient. At patient discharge from the ICU or death (within 30 days), anatomic injury diagnoses with corresponding Abbreviated Injury Scale (AIS) codes, and the ISS were recorded from medical records. The AIS was coded according to the 1998 updated classification [9] by local research assistants using medical, radiological and surgical reports. Local ICU physicians reviewed all problematic cases.

### End points

The main outcome measurement was the vital status at 30 days or at ICU discharge, if discharge occurred within the first 30 days. A secondary outcome was 72-h mortality.

### Statistical methods

Given their non-Gaussian distribution, quantitative variables were *a priori *categorized as follows: GCS score (<8, 8 to 13, >13), ISS (<25, 25 to 34, >34), systolic arterial blood pressure (<90, 90 to 110, >110 mmHg), SpO_2 _(<90, 90 to 95.9, ≥96%), respiratory rate (<10, 10 to 29, >29 minutes^-1^). Descriptive characteristics were expressed as percentages, or means with standard deviations (SD), or medians with interquartile range (IQR). Univariate comparisons between groups were performed using chi-square tests or Fisher exact tests, when appropriate, for qualitative variables, and using Kruskal-Wallis tests for quantitative variables. Multivariable analyses were performed using logistic regression models, where the outcomes (30-day mortality and 72-h mortality) were introduced as the dependent variables. Independent variables were: pre-hospital management (SMUR/non-SMUR), age, sex, injury severity score, systolic blood pressure, SpO_2_, respiratory rate, GCS score (model 1); and secondly, hospital of first admission or hospital admission delay (model 2). Interaction terms between SMUR/non-SMUR management and other independent variables were systematically tested. As none were significant, they were dropped from the final model. The Hosmer-Lemeshow test was used to check model goodness-of-fit. The significance level was *P *< 0.05. The Coordination Centre performed all analyses using SAS™ version 9.1 (SAS Institute Inc, Cary, NC, USA).

## Results

The 2,703 patients in the study sample comprised 2,063 men (76%) and 640 women (24%) with a mean age of 41.1 years (SD: 18.0 years). The median ISS was 25 (IQR: 18 to 34). Pre-hospital management was performed by SMUR for 2,513 patients (93%) and by fire brigades for 193 patients (7%). In comparison with non-SMUR patients, SMUR patients were significantly younger and more often directly referred to university hospitals (Table 1). SMUR patients were less frequently admitted to the first hospital within the first hour, and more frequently to an ICU within the first three hours after the accident. For the 2,015 patients (1,911 SMUR and 104 non-SMUR patients) with available information, the median time spent on the scene (IQR range) was significantly higher in SMUR patients (46 minutes, IQR: 30 to 68 minutes) than in non-SMUR patients (18 minutes, IQR: 13 to 27 minutes, *P *< 0.001). The median transport time was also higher in SMUR patients (54 minutes, IQR: 35 to 79 minutes) than in non-SMUR patients (40 minutes, IQR: 25 to 65 minutes, *P *< 0.001). SMUR patients were more frequently victims of traffic accidents than were non-SMUR patients. The type of pre-hospital management was not significantly related to gender, accident time or accident day.

**Table 1:** Patients' characteristics and accident circumstances among patients with severe blunt trauma according to pre-hospital management

	Pre-hospital management		*P*-value
	Non-SMUR (*n *= 190); n (%)	SMUR (*n *= 2513); n (%)	
Sex			0.16
Male	153 (81%)	1,910 (76%)	
Female	37 (19%)	603 (24%)	
			
Age *			0.015
18 to 29 y	51 (27%)	915 (36%)	
30 to 54 y	82 (43%)	1,039 (41%)	
55 to 69 y	31 (16%)	338 (13%)	
≥70 y	26 (14%)	219 (9%)	
			
First hospital of admission			<0.001
General hospital	118 (62%)	533 (21%)	
University hospital	72 (38%)	1,980 (79%)	
			
Delay to hospital admission			<0.001
<1 h	88 (46%)	340 (14%)	
1 to 3 h	85 (45%)	1,845 (73%)	
≥3 h	17 (9%)	328 (13%)	
			
Delay to ICU admission			<0.001
<1 h	29 (16%)	168 (7%)	
1 to 3 h	33 (18%)	1,478 (61%)	
≥3 h	120 (66%)	777 (32%)	
			
Accident type *			<0.001
Traffic accident	82 (43%)	1,595 63%)	
Pedestrian	11 (13%)†	181 (11%)†	
Bicyclist/motorcyclist	39 (48%)†	579 (36%)†	
Motor vehicle driver/passenger	32 (39%)†	832 (52%)†	
Other accidents	108 (57%)	917 (37%)	
Home/leisure/sport	85 (79%)†	717 (78%)†	
Occupational	12 (11%)†	172 (19%)†	
Miscellaneous (attack, suicides...)	10 (9%)†	27 (3%)†	
			
Accident time *			0.21
6 a.m to 11 p.m	119 (81%)	1881(85%)	
11 p.m to 6 a.m	28 (19%)	337 (15%)	
			
Accident day*			0.42
Working day	120 (63%)	1664 (66%)	
Weekend	69 (37%)	843 (34%)	

* Data were missing in a few patients for age (*n *= 2), accident type (*n *= 1), traffic accident victim condition (*n *= 4), accident day (*n *= 7) and in 338 patients for accident time

† Percentages calculated with either the number of traffic accidents or the number of other accidents as denominator

SMUR: Service Mobile d'Urgences et de Réanimation

As indicated in Table 2, an intravenous line was inserted in almost all SMUR patients and a large proportion of them received either crystalloids (71%) or colloids (47%). About half of the patients had tracheal intubation and were given artificial ventilation, whereas a smaller proportion (12%) received vasopressors in the pre-hospital phase. Very few patients received blood-derived products or required chest tube insertion. The GCS score had only a marginal influence on fluid administration. Although tracheal intubation and mechanical ventilation significantly decreased along with GCS score (*P *< 0.001), their use remained relatively frequent among patients with GCS score >13 (14.1% and 13.5%, respectively).

**Table 2:** Description of pre-hospital life-sustaining treatments among SMUR patients (*n *= 2513)

	All patients		By GCS* score		
	n/N†	%	<8%	8 to 13%	>13%
Venous line	2,400/2,431	98.7	99.8	98.8	97.9
Crystalloids	1,690/2,386	70.8	72.4	69.1	70.6
Colloids	1,119/2,385	46.9	54.9	37.8	45.1
Mannitol	84/2,385	3.5	8.5	2.4	0.3
Catecholamines	284/2,456	11.6	22.1	8.7	5.2
Tracheal intubation	1,258/2,484	50.6	98.0	54.1	14.1
Mechanical ventilation	1,222/2,484	49.2	97.5	53.4	13.5
Blood products	81/2,463	3.3	3.7	3.1	2.8
Chest tube	45/2,450	1.8	2.0	1.5	1.7

* Glasgow coma scale

† n, number of patients with specified treatment; N, number of patients with available information about specified treatment. SMUR, Service Mobile d'Urgences et de Réanimation

Among SMUR patients, 74 presented with cardiac arrest during transport and were alive upon arrival at the hospital. Among these, 14 patients (19%) survived to post-trauma Day 30, but only 3 patients made a good neurological recovery. These patients were excluded from subsequent analyses for two reasons. As mentioned above, because of the French law that imposes a systematic call to SMUR in cases of cardiac arrest during transport, we were unable to distinguish between patients initially managed by fire brigades and those initially managed by SMUR. Secondly, we had incomplete information about the clinical status and injury assessment for the majority of these patients.

The initial physiological status, GCS score and ISS according to type of pre-hospital management are compared in Table 3. In comparison with patients transported by fire brigades, SMUR patients had a poorer initial status. They presented a lower GCS score (*P *< 0.001), SpO_2 _(*P *= 0.052), a higher ISS (*P *< 0.001) and a higher frequency of abnormal pupils (*P *< 0.001).

**Table 3:** Initial assessment and injury severity score according to pre-hospital management

	All patients		Pre-hospital management				*P*-value
	(*n *= 2,629)		Non-SMUR (*n *= 190)		SMUR (*n *= 2,439)		
	N	(%)	n	(%)	n	(%)	
GCS*							<0.002
<8	775	(30.3)	26	(17.3)	749	(31.2)	
8 to 13	566	(22.2)	35	(23.3)	531	(22.1)	
≥14	1,213	(47.5)	89	(59.3)	1,124	(46.8)	
							
Abnormal pupils*							0.028
No	2,122	(84.2)	148	(90.2)	1,974	(83.8)	
Yes	398	(15.8)	16	(9.8)	382	(16.2)	
							
SpO2* (%)							0.052
<90	309	(12.3)	10	(6.2)	299	(12.7)	
90 to 95.9	480	(19.1)	33	(20.5)	447	(19.0)	
≥96	1,723	(68.6)	118	(73.3)	1,605	(68.3)	
							
Respiratory rate (min^-1^) *							0.18
<10	32	(1.2)	0	(0.0)	32	(1.3)	
10 to 29	2,421	(93.1)	181	(95.8)	2,246	(92.9)	
≥30	148	(5.7)	8	(4.2)	140	(5.8)	
							
Systolic blood pressure* (mm Hg)							0.38
<90	263	(10.3)	15	(8.9)	248	(10.4)	
90 to 109	447	(17.5)	24	(14.3)	423	(17.8)	
≥ 110	1,839	(72.1)	129	(76.8)	1,710	(71.8)	
							
Mean arterial blood pressure* (mm Hg)							0.36
<60	176	(6.9)	10	(5.8)	166	(7.0)	
60 to 90	1,090	(42.8)	66	(38.6)	1,024	(43.1)	
≥90	1,281	(50.3)	95	(55.6)	1,186	(49.9)	
							
Injury severity score							<0.001
<25	1,068	(40.6)	106	(55.8)	962	(39.4)	
25 to 34	992	(37.7)	70	(36.8)	922	(37.8)	
≥35	569	(21.6)	14	(7.4)	555	(22.8)	

* Data were missing in some patients for GCS scale (*n *= 75), abnormal pupils (*n *= 109), SpO_2 _(*n *= 117) systolic blood pressure (*n *= 80), mean blood pressure (*n *= 82) and respiratory rate (*n *= 28)

GCS, Glasgow Coma Scale; SMUR, Service Mobile d'Urgences et de Réanimation.

(Exclusion of 74 patients with cardiac arrest in the pre-hospital phase).

Up until hospital discharge (within 30 days after the accident), the death rate was comparable in patients transported by fire brigades (29 deaths, 15%) and SMUR patients (407 deaths, 17%, *P *= 0.61) (Table 4). Stratification on the GCS score revealed that the risk of death was systematically lower among SMUR patients than in patients transported by fire brigades, although the between-group difference only reached the level of significance among patients with a GCS score ≥14 (*P *= 0.025). A significantly better prognosis was also observed among SMUR patients with an ISS <25 (*P *= 0.002).

**Table 4:** Death rate before ICU discharge (within 30 days) according to pre-hospital management and selected characteristics (exclusion of 74 patients with cardiac arrest in the pre-hospital phase)

		Number of deaths (%) by pre-hospital management		
	Total	Non-SMUR *n *= 190	SMUR *n *= 2439	*P*-value
All deaths	436 (17%)	29 (15%)	407 (17%)	0.61
				
First hospital admission				
General hospital (*n *= 642)	107 (17%)	22 (19%)	85 (16%)	0.52
University hospital (*n *= 1,987)	329 (17%)	7 (10%)	322 (17%)	0.11
				
Delay to hospital admission				
<1 (*n *= 413)	68 (16%)	14 (16%)	54 (17%)	0.87
1 to 3 (*n *= 1,874)	309 (16%)	12 (14%)	297 (17%)	0.55
≥3 (*n *= 342)	59 (17%)	3 (18%)	56 (17%)	1
				
Delay to ICU admission				
<1 (*n *= 186)	27 (15%)	3 (10%)	24 (15%)	0.77
1 to 3 (*n *= 1,462)	245 (17%)	3 (9%)	242 (17%)	0.23
≥3 (*n *= 886)	153 (17%)	23 (19%)	130 (17%)	0.55
				
GCS				
<8 (*n *= 775)	279 (36%)	10 (38%)	269 (36%)	0.79
8 to 13 (*n *= 566)	76 (13%)	7 (20%)	69 (13%)	0.30
≥14 (*n *= 1,213)	73 (6%)	10 (11%)	63 (6%)	0.032
				
Injury Severity Score				
<25 (*n *= 1,068)	61 (6%)	13 (12%)	48 (5%)	0.002
25 to 34 (*n *= 992)	192 (19%)	14 (20%)	178 (19%)	0.89
≥35 (*n *= 569	183 (32%)	2 (14%)	181 (33%)	0.24

Analysis performed among 2,629 patients without cardiac arrest during the pre-hospital phase. GCS, Glasgow Coma Scale; OR, odds ratio; SMUR, Service Mobile d'Urgences et de Réanimation

Since SMUR patients presented with a more severe initial status, the relationship between the type of pre-hospital management and the risk of death was first adjusted for the GCS score, the ISS and the main initial physiological variables. The risk of death at 30 days was significantly lower (OR: 0.55, 95% CI: 0.32 to 0.94, *P *= 0.03) in SMUR patients than in non-SMUR patients (Table 5, model 1). Increasing age, high ISS, low GCS score, low initial systolic blood pressure and SpO_2 _<90% were all significant risk factors for death, whereas gender and initial respiratory rate were not (model 1). The association between pre-hospital management and 30-day mortality was not modified by further adjustment for the hospital of first admission (OR: 0.55, 95% CI: 0.31 to 0.98, *P *= 0.043). A short delay to hospital admission (less than one hour) was an independent risk factor for death, and only marginally affected the association with pre-hospital management (Table 5, model 2). In addition, results were not modified by adjustment for delay of ICU admission (OR: 0.52, 95% CI: 0.29 to 0.91, *P *= 0.022). Patients admitted to hospital after a more than one hour delay were more often intubated (53% vs. 32%, *P *< 0.001), ventilated (52% vs. 31%, *P *< 0.001) and more often received catecholamines (12% versus 8%, *P *= 0.048) than patients admitted more quickly.

**Table 5:** Association between physician pre-hospital management and death before ICU discharge (within 30 days) in multivariable analysis*

	Model 1			Model 2		
	OR	95% CI	*P*-value	OR	95% CI	*P*-value
Pre-hospital management						
Non-SMUR	1	-		1	-	
SMUR	0.55	0.32 to 0.94	0.030	0.62	0.35 to 1.10	0.10
						
Age (for 10 y variation)	1.48	1.38 to 1.59	<0.001	1.48	1.38 to 1.59	<0.001
						
Sex						
Female	1	-		1	-	
Male	0.95	0.71 to 1.27	0.75	0.95	0.71 to 1.27	0.72
						
Injury Severity score						
≤24	1	-		1	-	
25 to 34	3.18	2.24 to 4.51	<0.001	3.26	2.29 to 4.63	<0.001
≥ 35	5.96	4.09 to 8.67	<0.001	6.01	4.13 to 8.77	<0.001
						
Systolic arterial blood pressure (mmHg)						
<90	1.60	1.10 to 2.32	0.015	1.60	1.10 to 2.34	0.014
90 to 109	1.29	0.91 to 1.81	0.15	1.28	0.91 to 1.81	0.16
≥110	1	-		1	-	
						
SpO_2 _(%)						
<90	1.44	1.02 to 2.03	0.036	1.46	1.04 to 2.06	0.029
90 to 95.9	0.84	0.60 to 1.17	0.30	0.82	0.58 to 1.15	0.25
≥96	1	-		1	-	
						
Respiratory rate (min^-1^)						
<10	1.23	0.43 to 3.51	0.70	1.18	0.41 to 3.37	0.76
10 to 29	0.96	0.56 to 1.66	0.89	0.96	0.55 to 1.66	0.89
≥30	1	-		1	-	
						
GCS						
≤7	8.52	6.14 to 11.8	<0.001	8.70	6.296 to 12.1	<0.001
8 to 13	2.52	1.72 to 3.68	<0.001	2.51	1.72 to 3.67	<0.001
≥14	1	-		1	-	
						
Delay to hospital admission (h)						
<1	Not entered	-		1.65	1.00 to 2.71	0.048
1 to 3		-		1.20	0.82 to 1.76	0.35
≥3		-		1	-	

*Analysis performed among 2,359 patients without cardiac arrest during the pre-hospital phase for whom all data were available.

GCS, Glasgow Coma Scale; OR, odds ratio; SMUR, Service Mobile d'Urgences et de Réanimation

The mortality rate at 72 h tended to be higher in SMUR patients than in non-SMUR patients (10.3% and 6.3%, respectively, *P *= 0.079). After adjustment for other prognostic factors, the impact of SMUR management on the risk of death at 72 h was not significant (OR = 0.77; 95% CI: 0.38 to 1.59; *P *= 0.49).

## Discussion

To our knowledge, this is the first large prospective study to examine the impact of SMUR vs. non-SMUR management of severe blunt trauma on mortality. This study revealed that SMUR management of severe blunt trauma in France was associated with an almost 50% reduction in 30-day mortality. SMUR management had no apparent benefit on 72-h mortality.

In accordance with previous studies, the present study confirmed the major effect on 30-day mortality of a low initial GCS score (<8) [10,11], low systolic arterial blood pressure (<110 mmHg) [12], high injury severity score [13,14] and initial hypoxemia [15]. As previously reported, respiratory rate was not an independent risk factor for death [16].

In contrast to the beneficial impact of SMUR on 30-day mortality, the risk of death at 72 h was not significantly reduced in our study. The lack of any association may be partly due to the small number of events. The known bimodal distribution of the probability of death in blunt trauma patients may also explain this finding. Patients with very severe injury will die regardless of the type of pre-hospital management [17]. Another explanation may be due to our definition of severe blunt trauma. Indeed, only SMUR patients and patients admitted to a university ICU within 72 h were considered in the study. As a result, the number of early in-hospital deaths for non-SMUR patients may have been under-estimated since 62% of these patients were first admitted to general hospitals.

In France, pre-hospital care provided by fire brigades consists of oxygen administration, immobilization, dressing and cardio-pulmonary resuscitation with a bag valve in cases of cardiac arrest. Only EPs are allowed to perform SMUR management based on a large range of therapeutic strategies. These strategies, used after initial medical assessment, may include careful use of fluid administration, small-volume resuscitation strategies, continuous vasopressor infusion if fluid resuscitation fails to restore arterial pressure, tracheal intubation for mechanical ventilation after rapid sequence induction and continuous infusion of sedatives and analgesic agents, mannitol in cases of suspected clinical intracranial hypertension, and chest tube insertion. As pointed out by some authors, the main disadvantage of intensive pre-hospital management concerns patients with haemorrhagic shock in whom pre-hospital blood administration may delay hospital admission and hospital haemostatic treatment [2].

Our findings on the beneficial impact of SMUR management on 30-day mortality should be discussed with regard to the results of the OPALS (Ontario Prehospital Advanced Life Support) Major Trauma Study. This study, which compared advanced life support (ALS) and basic life support (BLS) performed by paramedics, found no significant difference between groups [18]. The population characteristics and trauma severity in the two studies were very similar. In contrast, the major outcome in the OPALS study was survival to hospital discharge, which is not strictly comparable to the outcome of the FIRST study. As pre-hospital ALS was performed by trained paramedics in the OPALS study, the spectrum of intensive care therapies was more limited than in the FIRST study.

The FIRST study suggests that the French SMUR system leads to more vigorous on-scene management than ALS provided by trained paramedics. First, the management of haemodynamic status is more intensive. As reported by another recent French study [19], an intravenous line was inserted in 99% of SMUR patients in the FIRST study compared to 63% of the ALS patients in the OPALS study [18]. According to French pre-hospital guidelines, a saline or colloid solution may be used for all patients on-scene with different blood pressure targets depending on whether patients have neurological injury or not. This early therapy in blunt trauma aims to limit excess fluid loading and later risks of multiple organ failure, acute lung injury and haematological complications [20]. In the FIRST study, around 47% of patients received colloid solution. The paramedics in the OPALS study did not have access to colloids and only 11.7% of the OPALS patients received intravenous fluid bolus therapy [18].

The influence of broad and early intubation on mortality is controversial [4]. Some studies suggested that it may be beneficial [21,22], whereas others did not [23,24]. Our study suggests that intensive pre-hospital airway management may explain the survival benefit for SMUR patients. In contrast to other countries, rapid sequence intubation for airway management is usual in France [25]. Nearly 50% of patients managed by EP were ventilated on-scene in the FIRST study, compared to under 7% intubated in the ALS group of the OPALS study [18]. In our study, indications for tracheal intubation and mechanical ventilation on-scene were not limited to patients with GCS scores <8 (97.5% ventilated patients) but extended to some patients with GCS scores between 8 and 13 (53.4% ventilated) and >13 (13.5% ventilated). This strategy, intended to increase the arterial oxygen level, is usually administered together with continuous infusion of sedatives and analgesic agents on-scene in order to decrease oxygen consumption [26]. Intensive airway management by EP possibly contributed to the reduction in acute respiratory distress syndrome (ARDS) and thus, to a decrease in 30-day mortality. On the basis of experimental studies [27-29], the use of continuous infusion of norepinephrine is suggested for sedated patients with hemorrhagic shock in order to avoid excess volume loading. This strategy, in association with frequent use of mechanical ventilation, may contribute to a decreased risk of ARDS [30] and in-hospital mortality [31].

The present study has the advantage of being prospective, based on a large sample of adult trauma patients consecutively recruited in university hospital ICUs located throughout France. Furthermore, the population was relatively homogenous since only patients with severe blunt trauma were included. However, our study also presents some limitations. The number of patients managed by fire brigades was low, limiting the statistical power of the study. The study was observational and did not allow any causal relationship to be established between the type of pre-hospital management and mortality. Clearly, the initial clinical status was more severe in SMUR than in non-SMUR patients, which reflects the efficiency of the French dispatching system. Differences in initial physiological status and injury severity between the two groups were taken into account in the outcome analysis, as well as the first admission hospital and the delay of first hospital admission or ICU admission. Our adjustment strategy did reveal the beneficial impact of SMUR, although this impact was not apparent in unadjusted analysis. Another limitation lies in our inability to control some potential confounding factors. For example, comorbidities were not recorded and information on time spent on the scene and transport time was available for only 76% and 55% of SMUR and non-SMUR patients, respectively. Furthermore, only patients directly or subsequently admitted to university hospitals were included. Thus, we cannot extrapolate our results to patients managed exclusively in general hospitals.

European pre-hospital management systems, particularly the French system, are controversial [2]. The main subject of debate is the increasing delay to hospital admission, diagnosis and actual salvage haemostasis induced by SAMU/SMUR intervention and on-scene management. In accordance with a recent study [32], the FIRST study showed that a short delay to hospital admission of less than one hour was an independent risk factor for death among patients with severe blunt trauma. The lower rate of tracheal intubation, ventilation, and vasopressor administration in patients rapidly admitted to hospital strongly suggests that EP involvement in starting resuscitation care early before hospital admission could be beneficial for patients with severe blunt trauma, as reported in other studies [33-35].

## Conclusions

This observational study suggests that, despite delayed hospital admission, SMUR management was associated with lower 30-day mortality after blunt trauma. The French SAMU/SMUR emergency system comes at a high cost to society. This cost should be balanced against the number of life years gained for trauma patients who are often in the youngest age range of the population. Clearly, our results need to be confirmed in a randomized trial, but such a trial would be very difficult to organize in France.

## Key messages

• The FIRST study is an epidemiological study designed to prospectively describe the management and care of severe blunt trauma in France.

• Severe blunt trauma patients may have medical pre-hospital management or only management by fire brigades, according to the French pre-hospital health organization.

• Medical pre-hospital management is associated with a significant reduction in 30-day mortality. (OR: 0.55, 95% CI: 0.32 to 0.94, *P *= 0.03)

• Despite a longer out of hospital time, the organization SAMU/SMUR ameliorates delays to university hospital (trauma centre level 1) admission leading to a positive impact on survival. (OR: 0.62, 95% CI: 0.62 to 1.10, *P *= 0.1)

## Abbreviations

AIS: abbreviated injury scale; ALS: advanced life support; ARDS: acute respiratory distress syndrome; BLS: basic life support; CI: confidence interval 95%; EP: emergency physician; FIRST: French Intensive care Recorded Severe Trauma (Study Group); GCS: Glasgow Coma Scale; IQR: interquartile range; ISS: injury severity score; MAP: mean arterial pressure; ml: milliliter; mmHg: mercury millimeter; OR: odds ratio; SAMU: Service d'Aide Médicale Urgente; SD: standard deviation; SMUR: Service Mobile d'Urgences et de Réanimation; SpO_2: _pulse oxymetry.

## Competing interests

The authors declare that they have no competing interests.

## Authors' contributions

YJM was involved in the study design, in the acquisition, analysis and interpretation of data, and wrote the first draft of the manuscript. GD, JC, DJ, MC and RF participated in the design of the study, in the acquisition of data and in the final revision of the manuscript. RB was involved in the initiation and design of the study, participated in the acquisition of data and contributed to the interpretation of data and final revision of the manuscript. BC participated in the design of the study and performed the statistical analysis. BKC was responsible for the logistic coordination of the study, was involved in the design of the study, in statistical analysis and interpretation of data and helped to draft the manuscript. FM initiated and coordinated the study and was involved at all steps of the study. All authors read and approved the manuscript.

## Supplementary Material

Additional file 1 **FIRST Study Group**. A full list of participants for the FIRST Study Group. Click here for file
