# Supplementary material for: Medical pre-hospital management reduces mortality in severe blunt trauma: a prospective epidemiological study
Source: Crit Care. 2011 Jan 20;15(1):R34. doi: 10.1186/cc9982 (PMC3222071; doi:10.1186/cc9982)
Supplement: Additional file 1 — FIRST Study Group. A full list of participants for the FIRST Study Group. [file cc9982-S1.DOCX]

The following investigators participated in the FIRST study group:

*Besançon*

Pr Annie BOILLOT, Dr Gilles BLASCO, Pr Emmanuel SAMAIN, Département d'Anesthésie Réanimation Chirurgicale, Pr Gilles CAPELLIER, Dr Thibault DESMETTRE, Dr Gabriel HAMADI, SAMU 25, CHU de Besançon-Hôpital Jean Minjoz.

*Dijon*

Pr Marc FREYSZ, Dr Jean-Michel YEGUIAYAN, Dr Christophe AVENA, Dr Sébastien ANDRE, Dr Philippe REVIRON Service d’Anesthésie Réanimation – SAMU 21; Dr Dalila SERRADJ, Service d’Accueil des Urgences. CHU de Dijon-Hôpital Général.

*Grenoble*

Dr Claude JACQUOT, Dr Céline GOURLE, Dr Julien BRUN, Dr Frédéric MONGENOT, Département d'Anesthésie Réanimation; Dr Elisabeth RANCUREL, Dr Bénédicte BOURGEOIS, Dr Isabelle FAVIER, SAMU 38, Dr François COPPO, Réanimation Neurochirurgicale, CHU de Grenoble-Hôpital de la Tronche.

*Lille*

Dr Patrick GOLDSTEIN, Dr Hervé COADOU, Dr Vincent MAREL, SAMU 59, Dr Delphine GARRIGUE, Dr Sandrine ROSENBERG, Service d'Accueil des Urgences; Dr POIDEVIN, Service d'Anesthésie Neurochirurgicale; Dr Bernard LEROY, Service d'Anesthésie Réanimation, Centre Hospitalier Régional et universitaire de Lille.

*Limoges*

Dr Dominique CAILLOCE, Dr Stéphanie SEBBAN, SAMU 87, Centre Hospitalier Régional et Universitaire de Limoges-Hôpital Dupuytren.

*Lyon*

Dr François ARTRU, Dr Frédéric DAILLER, Dr Thomas LIEUTAUD, Dr Carole BODONIAN, Dr Jacqueline CONVERT, SIPO – U800, Hôpital Neurologique et Neurochirurgicale Pierre Wertheimer, Bron.

Dr Sarah LORGE, SAMU 69; Dr Philippe RAGUE, Dr Marie Christine LAPLACE, Dr Carine DELALEU-RAGUE, Dr Jean-Stephane DAVID, Dr Laure BESSON, Pr. Pierre Yves GUEUGNIAUD, Pôle Urgence et Réanimation Médicale-SAMU, Groupe hospitalier Edouard Herriot, Lyon.

*Marseille*

Dr François ANTONINI, Pr. Claude MARTIN, Service Anesthésie Réanimation, Hôpital Nord, Marseille.

*Nantes*

Dr Antoine ANDRE, Dr Jean-Pierre GOURAUD, SAMU 44; Pr. Michel PINAUD

Dr Philippe CHAMPIN, Pôle Anesthésie Réanimation; Dr Dominique DEMEURE, Dr Pierre Joachim MAHE, Réanimation chirurgicale, Centre Hospitalier Universitaire – Hotel Dieu, Nantes

*Nîmes*

Pr Jean Yves LEFRANT, Dr Sophie LOUVARD, Pr. J.E. DE LA COUSSAYE, Dr Pierre GERAUD CLARET, Dr Aurélie DARDALHON, Division Anesthésie-Réanimation-Douleur-Urgence, Centre Hospitalier Universitaire de Montpellier-Nîmes, Nîmes

*Paris – Ile de France*

Pr Jacques DURANTEAU, Dr Christian LAPLACE, Dr Gaëlle CHEISSON, Dr Bernard VIGUE, Dr Pierre-Etienne LEBLANC, Dr Olivier HUET, Dr Catherine RACT, Unité de Réanimation Chirurgicale CHU Bicêtre, Le Kremlin-Bicêtre.

Pr Bruno RIOU, Dr. Danielle SARTORIUS, Dr. Yan ZHAO, Service d’Accueil des Urgences, Pr Olivier LANGERON, Dr Frédéric MARMION, Dr Sabine ROCHE, Dr Julien AMOUR, Dr Armelle NICOLAS ROBIN, Département D’Anesthésie Réanimation, Groupe Hospitalier la Pitié Salpétrière, Paris.

Dr Caroline TELION, Dr Jean-Sébastien MARX, Dr Yaël ICHAY, Dr Kim AN, Dr Benoit VIVIEN, Pr Pierre CARLI, SAMU 75, Hôpital Necker, Paris.

*Poitiers*

Dr Jean Yves LARDEUR, Dr Etienne QUOIRIN, Service des Urgences, Dr Fatima RAYEH, Pr. Olivier MIMOZ, Réanimation Chirurgicale Polyvalente, CHU - Hôpital Jean Bernard, Poitiers.

*Coordination center for data monitoring and statistical analysis* - Centre d’Investigation clinique - Epidémiologique clinique du CHU de Dijon (INSERM CIE 01), Dijon, France: Pr Claire BONITHON-KOPP (coordinator), Pr Christine BINQUET (head statistician), Elodie GAUTIER and Sandrine VINAULT (statisticians/data managers), Alexandra FELIN (study monitor). Local research assistants: Nathalie BERGER (Nantes, Poitiers), Brigitte LAFOND and Françoise CASANO (Lyon, Marseille, Nîmes), Carine PIATEK (Lille, Paris), Alexandra FELIN (Grenoble, Besançon, Dijon).
